# Loss of *Foxd4* Impacts Neurulation and Cranial Neural Crest Specification During Early Head Development

**DOI:** 10.3389/fcell.2021.777652

**Published:** 2022-02-01

**Authors:** Riley McMahon, Tennille Sibbritt, Nadar Aryamanesh, V. Pragathi Masamsetti, Patrick P. L. Tam

**Affiliations:** ^1^ Embryology Research Unit, Children’s Medical Research Institute, Sydney, NSW, Australia; ^2^ School of Medical Sciences, Faculty of Medicine and Health, The University of Sydney, Darlington, NSW, Australia

**Keywords:** Foxd4, head development, anterior neuroectoderm, neural tube defects, cranial neural crest

## Abstract

The specification of anterior head tissue in the late gastrulation mouse embryo relies on signaling cues from the visceral endoderm and anterior mesendoderm (AME). Genetic loss-of-function studies have pinpointed a critical requirement of LIM homeobox 1 (LHX1) transcription factor in these tissues for the formation of the embryonic head. Transcriptome analysis of embryos with gain-of-function LHX1 activity identified the forkhead box gene, *Foxd4,* as one downstream target of LHX1 in late-gastrulation E7.75 embryos. Our analysis of single-cell RNA-seq data show *Foxd4* is co-expressed with *Lhx1* and *Foxa2* in the anterior midline tissue of E7.75 mouse embryos, and in the anterior neuroectoderm (ANE) at E8.25 alongside head organizer genes *Otx2* and *Hesx1*. To study the role of *Foxd4* during early development we used CRISPR-Cas9 gene editing in mouse embryonic stem cells (mESCs) to generate bi-allelic frameshift mutations in the coding sequence of *Foxd4*. In an *in vitro* model of the anterior neural tissues derived from *Foxd4*-loss of function (LOF) mESCs and extraembryonic endoderm cells, expression of head organizer genes as well as *Zic1* and *Zic2* was reduced, pointing to a need for FOXD4 in regulating early neuroectoderm development. Mid-gestation mouse chimeras harbouring *Foxd4*-LOF mESCs displayed craniofacial malformations and neural tube closure defects. Furthermore, our *in vitro* data showed a loss of FOXD4 impacts the expression of cranial neural crest markers *Twist1* and *Sox9*. Our findings have demonstrated that FOXD4 is essential in the AME and later in the ANE for rostral neural tube closure and neural crest specification during head development.

## Introduction

The head is the first major body part to form immediately following gastrulation in vertebrate embryos, arising from the anterior germ layer tissues. In mice, the anterior-posterior axis is polarized by the visceral endoderm cells that are relocated from the distal tip of the epiblast to become the anterior visceral endoderm (AVE) Perea-Gómez et al. (1999). The AVE is involved in inducing anterior neuroectoderm (ANE) identity in the anterior epiblast prior to the anterior mesendoderm (AME) tissue re-enforcing the identity (Thomas and Beddington, 1996; Kimura et al., 2000). The role of the AME tissue at late gastrulation is to antagonize the posteriorizing signaling activity such as WNT and BMP (Arkell and Tam, 2012). Knock-out mouse models for key transcription factors *Lhx1* and *Foxa2,* expressed in both the AVE and AME, result in severe truncation of the embryonic head (Ang and Rossant, 1994; Shawlot and Behringer, 1995). The lack of *Lhx1* and *Foxa2* activity disrupts the formation of AME and notochord tissues, resulting in the loss of head precursor tissues (Kinder et al., 2003). Earlier work has identified many of the downstream targets of LHX1 in the AME are involved in the suppression of WNT signaling including *Gsc*, *Dkk1* and *Cer1* (Fossat et al., 2015; McMahon et al., 2019). To further study the potential target of LHX1 in the E7.75 mouse embryos, a conditional *Lhx1*-LOF model was used to identify the genes that are down-regulated with *Lhx1*-LOF (Sibbritt et al., 2018). Genes identified as potential targets of LHX1 include head organizer transcription factors *Hesx1* and *Otx2*, as well as *Foxd4*. *Hesx1* and *Otx2* are both expressed in the AME and ANE of early-head-fold stage (E7.75) embryos, *Hesx1* expression is then up-regulated in the forebrain, whilst *Otx2* is expressed in the midbrain of neurulation stage embryos (Simeone et al., 1993; Hermesz et al., 1996). Knockout of either genes resulted in a truncated head at early-organogenesis stage (Matsuo et al., 1995; Martinez-Barbera et al., 2000).

*Foxd4* is a member of the forkhead/winged helix-box transcription factors that is expressed in the notochord, AME and ANE of mouse embryos (Kaestner et al., 1995). *Foxd4* has been previously identified as a downstream target of *Foxa2* and *Otx2*. In E7.75 embryos lacking *Foxa2* activity, *Foxd4* was only expressed in the anterior neurectoderm, and missing in the AME (Tamplin et al., 2008). Conversely, in E7.75 *Otx2* ^−/−^ embryos, the expression of *Foxd4* was restricted to the distal AME and absent from the ANE (Rhinn et al., 1998). *Foxd4* is highly conserved between vertebrate species including humans, mice, frogs and zebrafish (Odenthal and Nüsslein-Volhard, 1998; Neilson et al., 2012). It contains an N-terminal acidic blob (AB) domain, a 100-amino acid forkhead domain and a C-terminal Engrailed homology (Eh1) domain. In *Xenopus* the AB domain was shown to activate neural precursor genes *Gem* and *Zic2*, whilst the Eh1 domain acted as a transcriptional repressor of genes responsible for neural differentiation (*Sox1, Irx*) (Neilson et al., 2012). FOXD4 is required in the transition of the mouse embryonic stem cell (mESCs) from pluripotency to neuroectoderm precursor cells (Sherman et al., 2017), though the function of FOXD4 *in vivo* has not been elucidated.

Our study explored the role FOXD4 plays in the anterior midline tissue and the ANE of the mouse embryo. We showed that *Foxd4* is co-expressed with head organizer genes *Lhx1* and *Foxa2* in the AME and notochord of late-gastrulation embryo, it is also co-expressed with *Otx2* and *Hesx1* in the ANE of the early somite stage embryo. Using *in vitro* and *in vivo* models generated using CRISPR-Cas9 gene edited mESCs, we showed that the loss of FOXD4 function resulted in a reduction in head organizer activity and the disruption of cranial neural crest (CNC) development. Furthermore, *Foxd4*-LOF chimeric embryos displayed dysmorphology of craniofacial structures and neural tube closure defects.

## Materials and Methods

### Cell Culture

R1 mESCs were grown on mouse embryonic fibroblasts (MEFs) and maintained in DMEM (Thermo Fisher Scientific), 12.5% heat inactivated fetal calf serum (Fisher Biotec), 10 mM *β*-mercaptoethanol, 1x non-essential amino acids (Thermo Fisher Scientific), 1X nucleosides (Merck) and 1X leukemia inhibitory factor (LIF). Cells were passaged at 70% confluency, 2–3 days after seeding onto pre-plated MEFs. For chimera generation, mESCs were maintained in 2i/LIF media (Ying et al., 2008) for at least 2 passages before use.

For *Lhx1* overexpression in chimeric embryos, two doxycycline inducible A2. loxCre mESC lines were used that either express a FLAG tagged wild-type *Lhx1* or tagged truncated *Lhx1* coding region lacking the functional LIM domains and homeodomain (Sibbritt et al., 2018).

Extraembryonic endoderm (XEN) cells were generated from blastocyst stage embryos as previously described (Niakan et al., 2013). ARC/s and *DsRed.T3* mice (from the Australian Animal Resources Centre) were maintained as homozygous breeding pairs. ARC/s females were crossed with *DsRed.T3* males, blastocyst stage embryos were collected and plated onto MEFs in TS cell medium; RPMI 1640 (Gibco), 20% fetal calf serum (Fisher Biotec), 2 mM l-glutamine (Gibco), *β*-mercaptoethanol, 1 mM sodium pyruvate (Gibco), 1% penicillin-streptomycin plus 24 ng/ml FGF4 (Sigma-Aldrich, cat. no. F8424) and 1 μg/ml heparin (Sigma-Aldrich, cat. no. H3393) for 20 days. The *dsRed* expressing XEN cells were then expanded on gelatin and maintained without FGF4 and heparin.

### CRISPR Editing

*Foxd4* edited mESCs were generated as described previously (Sibbritt et al., 2019). Guide RNAs targeting the N-terminal region of *Foxd4* were designed using (Benchling [Biology Software], 2021) (gRNA 1: 5′-CAG​TCC​TCT​AAG​TTC​CGA​CC, gRNA 2: 5′-GGA​GCG​ATC​CCT​GCA​GAG​GC) and ligated into pSpCas9(BB)-2A-Puro (PX459) V2.0 (a gift from Feng Zhang (Ran et al., 2013)). To induce editing, 5 × 10^6^ R1 mESCs were electroporated with 2.5 µg of plasmid DNA and plated onto mouse embryonic fibroblasts (MEFs) for 24 h before puromycin selection for 48 h. Individual clones were expanded on MEF coated plates and genotyped for correct edits in the *Foxd4* coding region.

The genotyping PCR products were gel purified and sub-cloned into the pGEM-T Easy Vector System (Promega) as per manufacturer’s protocol. At least 10 plasmids from each cell line were Sanger sequenced to identify mutations in each allele.

### Neuruloid Differentiation

Assemblies of mESCs and XEN cells (neuruloids) were generated as described previously (Bérenger-Currias et al., 2020) with some modifications. Approximately 2.5 × 10^6^ mESCs and 0.5 × 10^6^ XEN cells were mixed and placed in each well of a 24-well plate on 400 µm Aggrewells (Stem Cell Technologies) with 2 ml of N2B27 media, and then spun at 400 g for 3 minutes. The cells were cultured in Aggrewells for 48 h, and next transferred to low adhesion plates on a shaking platform with a 24-h pulse of 3 µm CHIR99021. CHIR99021 was then removed, and neuruloids were collected after a further 24 h of culture for RNA preparation and whole mount immunofluorescence microscopy.

### Neural Precursor Differentiation

Neural precursor differentiation of mESCs was initiated using embryoid bodies (EBs) as previously described (Varshney et al., 2017; Fan et al., 2021). After 4 days of differentiation, EBs were collected and plated on laminin (5 μg/ml) coated tissue culture plates in N2B27 media for a further 4 days of culture, then collected for RNA preparation or fixed in 4% paraformaldehyde for immunofluorescence imaging.

### Chimera Production

Chimeras were generated as previously described (Sibbritt et al., 2019; Fan et al., 2021). Briefly, ARC/s females were crossed with *Ds. RedT3* stud males, at E2.5 the uteri and oviducts were flushed to collect 8-cell stage embryo collection. 13–15 mESCs were injected per 8-cell *DsRed.T3* embryo, which were incubated overnight. 10 to 12 injected blastocyst-stage embryos were transferred to each E2.5 pseudo-pregnant ARC/s female recipient. E8.0—E11.5 embryos were collected 5–8 days after embryo transfer and imaged immediately on the Zeiss SteREO Lumar. V12 stereomicroscope to determine relative contribution of *dsRed* host cells versus injected mESCs. Relative intensity of dsRed.T3 fluorescence of each chimeric embryo was measured using ImageJ. The mean fluorescence of the dsRed.T3 channel was collated for each embryo and the background signal was subtracted. The mean fluorescence value was then displayed relative to embryo without ESC contribution at each stage. Animal experimentations were performed in compliance with animal ethics and welfare guidelines stipulated by the Children’s Medical Research Institute/Children’s Hospital at Westmead Animal Ethics Committee under protocol number C346.

### Immunofluorescence Imaging

Whole-mount immunostaining of chimeric embryos was performed as described in Fan et al. (2021), while immunostaining of neuruloids was performed as described in Dekkers et al. (2019). A list of antibodies and concentrations used are outlined in Supplementary Table S2. Embryos and neuruloids were imaged using Zeiss Cell Observer Spinning Disk Confocal Microscope. Three-dimensional images of the samples were produced using optical slices and tiling. Zeiss Zen microscopy analysis software was used to collapse the confocal stacks and stitch together tiles to generate maximum intensity projection (MIP) images.

Immunofluorescence imaging of neural precursor cells on glass cover slips was performed as described in Sibbritt et al. (2018) and imaged using the Zeiss Axio Imager M1 microscope.

### RT-qPCR

RNA was extracted using the RNeasy Mini Kit (Qiagen) for cells and Rneasy Micro Kit (Qiagen) for embryos, according to manufacturer’s protocol. cDNA was synthesised from 1 µg of RNA (or 0.3 µg for E8.0 embryos) using the SuperScript III First-Stand Synthesis System (Invitrogen, Cat. No. 18080-051) as per the manufacturer’s protocol, using random hexamers to prime the single-stranded RNA. Unless otherwise stated, quantitative PCR (qPCR) primers were designed using Primer-BLAST to span exon junctions of the functional mRNA transcript (all qPCR primers are listed in Supplementary Table S1). PowerUp SYBR Green PCR Master Mix (Thermo Fisher Scientific) and 0.4 µM of both forward and reverse primers were made to a total volume of 10uL PCR reaction. Samples were loaded into a 384 well plate (Thermo Fisher Scientific) and run on the QuantStudio 6 Flex Real-Time PCR System (Applied Biosystems). All reactions were performed in technical triplicates, relative gene expression was calculated using the comparative CT method, normalised to the housekeeping genes, *Actb* or *Ubc.*

Statistical significance was determined using an unpaired, two-tailed Student’s t-test, assuming unequal variances for single comparisons. *p* values were obtained relative to wild-type cells/chimeras if not indicated otherwise. Differences were considered significant if the *p < 0.05, **p < 0.01, ***p < 0.001, ****p < 0.0001.

### Bioinformatics Analysis of Single-Cell RNA-Seq Data

The processed read counts and metadata from Pijuan-Sala et al. (2019) were downloaded from https://github.com/MarioniLab/EmbryoTimecourse 2018. Reads were converted to a *Seurat* object and quality control of scRNA-seq data was performed with the *Seurat* package version 4.0.0 (Stuart et al., 2019) in *R* version 4.0.3. The data consisted of 29,452 genes with 139,331 single cells. The *scater/Bioconductor* package (McCarthy et al., 2017) was used to create QC metrics for the genes of interest. The *dittoSeq* package/Bioconductor (Bunis et al., 2020) was used for visualization of reduced dimension plots. Hierarchical cluster analysis was performed on a subset of cells expressing *Foxd4* for each stage using *hclust* (Müllner, 2013) with parameters “complete” method and “Euclidean” distance.

## Results

### *Lhx1*, *Foxa2* and *Otx2* Are Co-expressed With *Foxd4* in Early Mouse Embryo

**FIGURE 1:**
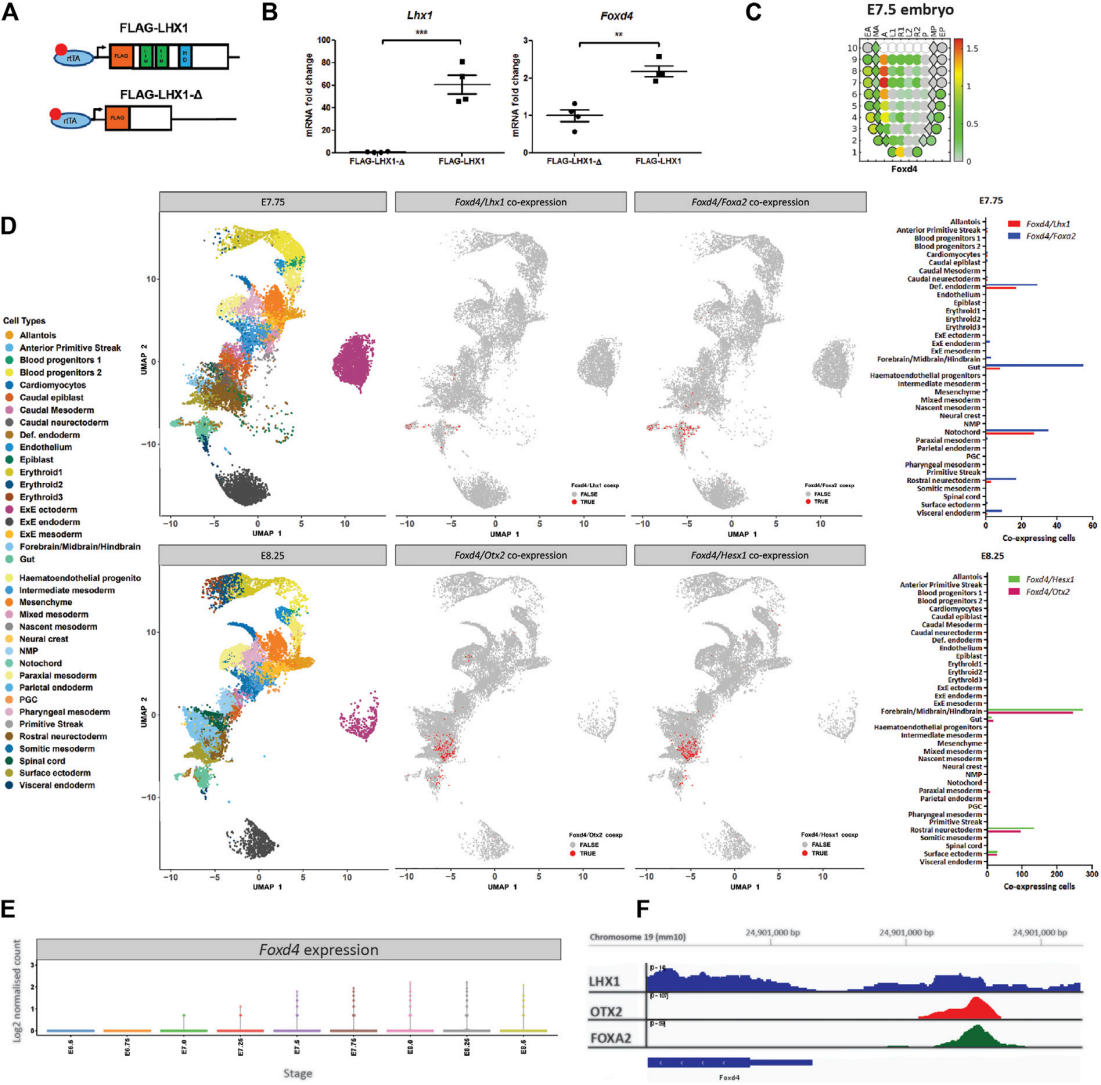
*Foxd4* is upregulated in an *Lhx1-*overexpressing embryo system and shows co-expression with *Lhx1*, *Foxa2* and *Otx2* in the late-to post-gastrulation mouse embryo. **(A)** Schematic representation of the *Hprt* locus of the A2lox.cre mouse ESCs (Iacovino et al., 2011) containing a tetracycline response element (TRE) followed by either FLAG-*Lhx1* wild-type coding sequence or FLAG-*Lhx1*-Δ mutant sequence lacking LIM domains and homeodomain. **(B)** RT-qPCR analysis of the expression of wild-type *Lhx1* and *Foxd4* (relative to *Actb*) in FLAG-*Lhx1*-Δ and FLAG-*Lhx1* E7.75 chimeras, respectively, with 24h of doxycycline treatment. Graphs are presented as mean ± SEM of four independent treatments (**p < 0.01, ***p < 0.001 by Student’s t test). **(C)** Corn plot shows the expression pattern of *Foxd4* in wild-type E7.5 embryos (Peng et al., 2019). High expression is seen in the anterior midline tissue and neurectoderm. The relative expression level is indicated by the color bar and the maximum relative expression in fragments per kilobase of transcript per million mapped reads (FPKM) is shown. **(D)** Uniform Manifold Approximation and Projection for Dimension Reduction (UMAP) for individual cells at E7.75 and at E8.25 (data from Pijuan-Sala et al., 2019). Colours represent the relevant cell types expressing the genes of interest. Co-expression of *Foxd4*/*Lhx1* and *Foxd4*/*Foxa2* are found in the notochord and definitive endoderm cell types in E7.75 embryo. Co-expression of *Foxd4* and *Otx2* as well as *Foxd4* and *Hesx1* in E8.25 embryos in the forebrain/midbrain/hindbrain and rostral neurectoderm cell types. **(E)** Expression of *Foxd4* in whole mouse embryos at E6.5 to E8.5. Log2 normalised count is presented. **(F)** University of California at Santa Cruz (UCSC) track view of ChIP-seq wiggle plot overlays showing enrichment of LHX1 (blue), OTX2 (red) and FOXA2 (green) at an upstream regulatory region of *Foxd4* on chromosome 19: 24,902,170–24,902,900 (mm10 genome).

A previous study has shown that conditional ablation of *Lhx1* in the epiblast reduced the expression of *Foxd4* in the anterior tissues of embryos (Sibbritt et al., 2018). Based on this finding, we hypothesise that the LHX1/FOXA2/OTX2 transcription factor complex drives the expression of *Foxd4* in the AME at E7.75 and in the ANE at E8.25. We validated our hypothesis that LHX1 can affect transcription of *Foxd4* using an *Lhx1*-overexpressing embryo model. Doxycycline inducible FLAG-*Lhx1* and FLAG-*Lhx1*-Δ (lacking functional domains) mESC lines were used to generate mouse chimeras (Figure 1A). Chimeras with high mESC contribution were collected at E7.75 following 24h of doxycycline treatment. Expression of wild-type *Lhx1* mRNA was 60-fold higher in FLAG-*Lhx1* vs FLAG-*Lhx1*-Δ (Figure 1B). A significant increase in *Foxd4* transcripts (Figure 1C) indicates that enhanced LHX1 activity affected the expression of *Foxd4*.

Using the publicly available eGastrulation spatial transcriptome dataset (Peng et al., 2019), we are able to investigate the location and relative level of *Foxd4* expression in the late gastrulation stage mouse embryo. The highest level of *Foxd4* expression can be seen in the anterior midline cell population and the neurectoderm (Figure 1C), consistent with previous *in situ* hybridization data (Tamplin et al., 2008). The spatial expression of *Foxd4* in late gastrulation mouse embryos overlaps with the known locations of genes that have been shown to be critical for embryonic head development such as *Lhx1*, *Foxa2*, *Otx2* and *Hesx1.*

To investigate the expression of these transcription factors at higher resolution, we used previously published single-cell RNA-seq data of wild-type mouse embryos (Pijuan-Sala et al., 2019). At E7.75 *Foxd4* is highly expressed in the notochord cell lineage (Supplementary Figure 1A), which gives rise to the midline mesendoderm tissues (Yamanaka et al., 2007). At this stage *Lhx1* and *Foxa2* share similar expression profiles (Supplementary Figure 1A) and our analysis identified co-expression of *Foxd4* with either *Lhx1* or *Foxa2* expressing cells in the notochord and definitive endoderm populations (Figure 1D). At E8.25 *Foxd4* is highly expressed in rostral neurectoderm and forebrain/midbrain/hindbrain cell populations. Similarly, the head organizer genes *Otx2* and *Hesx1* are expressed in these cell populations (Supplementary Figure 1A). Our analysis highlighted several groups of cells that share co-expression of *Foxd4, Otx2* and *Hesx1* at E8.25 (Figure 1D). *Foxd4* expression in the whole embryo is increasing at E7.5 and peaks at E8.0 (Figure 1E).

To elucidate if these transcription factors bind to the regulatory region of the *Foxd4* locus in mouse cells we retrieved the binding data from publicly available ChIP-seq dataset. LHX1 ChIP-seq data in differentiated P19 carcinoma cells shows a low confidence peak ∼1k bp upstream of the *Foxd4* transcriptional start site (TSS) (Figure 1F) (Costello et al., 2015). ChIP-seq data of OTX2 in epiblast like-cells (Buecker et al., 2014) and FOXA2 in mesendoderm cells (Cernilogar et al., 2019) show high confidence peaks in the same locus on chromosome 19: 24,902,170–24,902,900 (mm10 genome). These data suggest the binding of a LHX1/OTX2/FOXA2 transcription factor complex upstream of the *Foxd4* TSS.

### CRISPR-Cas9 Editing of *Foxd4* Coding Region Disrupts the Transcriptional Program of Anterior Epiblast *in vitro*

**FIGURE 2:**
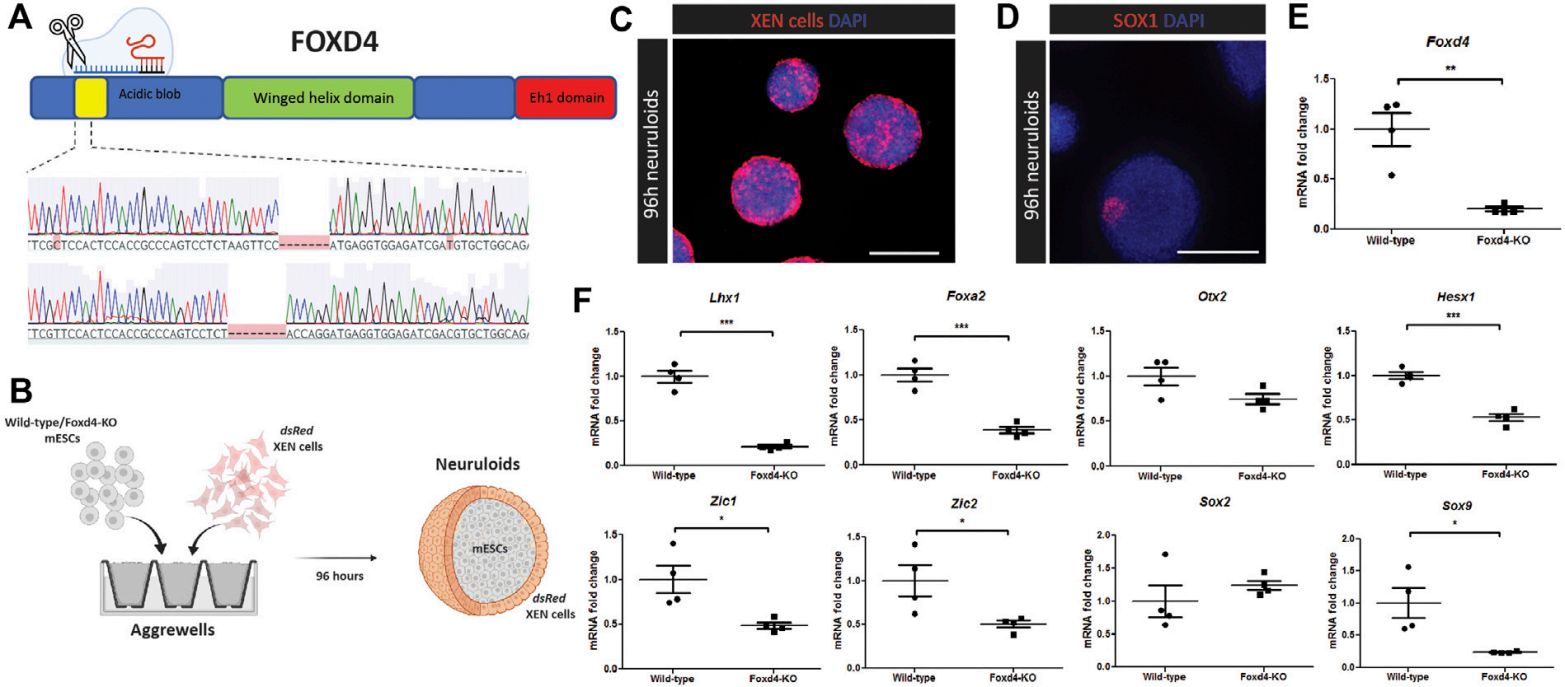
CRISPR-Cas9 editing of *Foxd4* coding region in mESCs results in reduced expression of anterior head and neural markers *in vitro*. **(A)** Schematic representation of CRISPR-Cas9 editing on *Foxd4*. The protein domains of FOXD4 encompasses the N-terminal DNA binding acidic blob domain, the winged helix domain and the C-terminal Eh1 domain. CRISPR-Cas9 gRNAs targeted the DNA sequence corresponding to the N-terminal acidic blob domain of *Foxd4,* resulting in two alleles with 7bp and 8bp deletions, respectively. **(B)** Schematic representation of an *in vitro* model of anterior late gastrulation mouse embryos (Bérenger-Currias et al., 2020). Wild-type or *Foxd4* ^*Δ7*/*Δ8*^ mESCs and *dsRed* XEN cells are co-cultured in aggrewells in N2B27 media. After 48 h, 3D organoids were moved to shaking culture with 3 μM of CHIR-99021 added for 24 h. The organoids were then collected after 96h of differentiation. **(C)** Neuruloids containing XEN cells expressing *dsRed* colonizing the outside of the neuruloids, and ESCs colonizing the core. Single z-stack. N = 4. Scale bar = 300 μm. **(D)** 96 h differentiated wild-type neuruloids show expression of early neural marker SOX1. Maximum intensity z projection. N = 4 replicate experiments. Scale bar = 300 μm. **(E)** RT-qPCR analysis of the expression of *Foxd4* (relative to *Ubc*) in Wild-type and *Foxd4* ^*Δ7*/*Δ8*^ neuruloids. **(F)** RNA expression of anterior tissue markers *Foxa2, Lhx1, Otx2* and *Hesx1*, neural precursor markers *Zic1, Zic2, Sox2* and neural crest marker *Sox9* (relative to *Ubc*). Data are presented as mean ± SEM of four independent experiments (*p < 0.05, **p < 0.01, ***p < 0.001 by Student’s t test).

In *Foxd4/5*, the *Foxd4* paralog in *Xenopus,* the AB domain has been shown to be a transcriptional activator of neural transcription factors (Neilson et al., 2012). We targeted this AB domain region in mESCs with CRISPR-Cas9 mediated genome editing (Figure 2A). Following screening, we chose clones with a bi-allelic frameshift mutation in the N-terminal region of *Foxd4,* at the beginning of the AB domain (Foxd4 ^*Δ7*/*Δ8*^, Foxd4^*Δ2*/*Δ2*^ (Figure 2A, Supplementary Figure 2A). We also used a different gRNA targeting the region between the AB and forkhead domain of *Foxd4,* to exclude off-target effects of CRISPR-Cas9 genome editing. Using the second gRNA we obtained a clone with a 2 bp deletion and a 1 bp insertion in respective alleles (Foxd4^*Δ2*/*Δ1*^ *,* Supplementary Figure 2D). Despite trying numerous antibodies from different manufacturers, we were unable to get a reliable signal to assay the expression of the predicted truncated FOXD4 protein in our knockout mESC line (data not shown).

To study the downstream genetic targets of FOXD4 during development, we used an *in vitro* model of the anterior epiblast (neuruloid) generated through the co-culture of mESCs and extraembryonic endoderm (XEN) (Figure 2B) (Bérenger-Currias et al., 2020). The XEN cells express genes that are highly expressed in the extraembryonic endoderm including *Foxa2*, *Sox17* and *Gata4*, but do not express pluripotency markers, *Oct4* and *Sox2* (Supplementary Figure 3A, B). The *dsRed* expressing XEN cells colonized the exterior portion of the neuruloid (Figure 2C, Supplementary Figure 3C), where they may act in a similar way to the anterior visceral endoderm population in the embryo. In contrast to embryoid bodies differentiated for the same period, we showed significantly higher expression of anterior markers *Otx2, Lhx1*, *Hesx1* as well as *Foxd4* (Supplementary Figure 3D). Wild-type neuruloids expressed early neurectoderm marker SOX1 in distinct regions of the neuruloids (Figure 2D, Supplementary Figure 3E).

Compared with neuruloids generated using wild-type mESCs, *Foxd4* ^*Δ7*/*Δ8*^ neuruloids had significantly reduced expression of *Foxd4* transcripts (Figure 2E). *Lhx1*, *Hesx1* and *Foxa2* transcripts were also significantly reduced (Figure 2F). This result indicates that FOXD4 is crucial for the appropriate specification of the precursor tissues to the embryonic head and notochord. Comparable to results seen in *Xenopus* (Neilson et al., 2012), knock-out of *Foxd4* caused the reduction in expression of neural ectodermal genes *Zic1* and *Zic2* (Figure 2F). Expression of the neural progenitor gene *Sox2* was not changed in *Foxd4* ^*Δ7*/*Δ8*^ neuruloids, whereas the transcripts of early neural crest cell (NCC) marker *Sox9* was significantly reduced, indicating a role for *Foxd4* in the establishment of the NCC population. Our analysis of scRNA-seq data from Pijuan-Sala et al. (2019) showed *Foxd4* is co-expressed with *Zic2* and *Sox9* but not *Zic1* at E7.75 and E8.25 in the ANE tissues (Supplementary Figure 1B).

### Mouse Chimeric Embryos Derived From *Foxd4-LOF* mESCs Display Neural Tube and Craniofacial Defects

**FIGURE 3:**
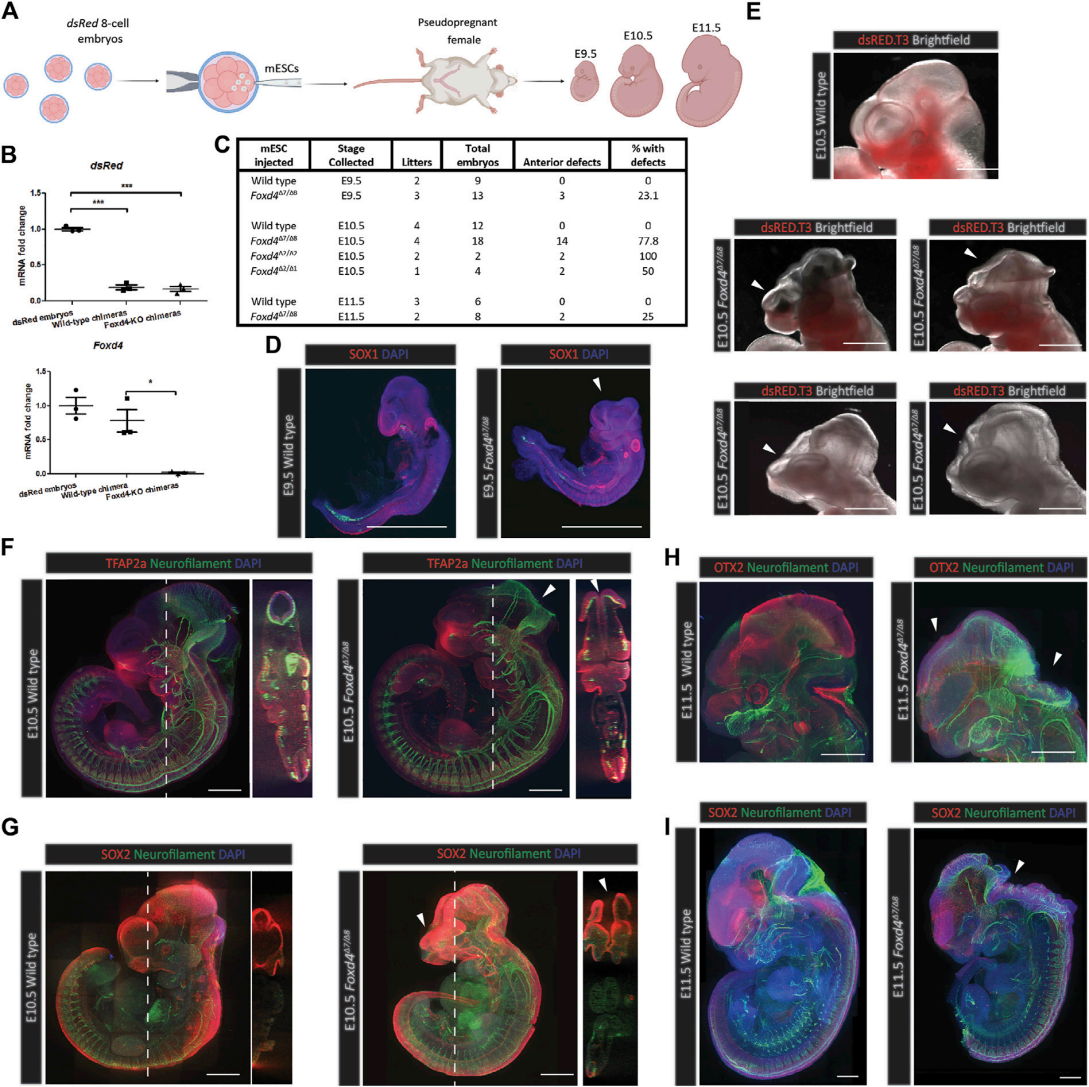
*Foxd4*-LOF mESC derived chimeras display neural tube closure defect and craniofacial dysmorphology. **(A)** Schematic representation of the generation of chimera mouse embryos. Chimeras were generated using wild-type or *Foxd4* ^*Δ7*/*Δ8*^ mESCs injected into 8-cell mouse embryo expressing *dsRed*. Following *in vitro* culture, the blastocyst stage embryos were transferred to the uteri of pseudo-pregnant female mice and were collected at various post-implantation time points up to E11.5. **(B)** Wildtype and *Foxd4* ^*Δ7*/*Δ8*^ mESC derived chimeras show similar contribution of mESCs through RT-qPCR analysis of *dsRed* mRNA compared to un-injected *dsRed* embryos (relative to *Ubc*) at E8.0. Expression of *Foxd4* (relative to *Ubc*) shows a significant reduction in *Foxd4* ^*Δ7*/*Δ8*^ chimeras vs wild-type chimeras. Data are presented as mean ± SEM of three independent embryos (***p < 0.001, *p < 0 0.05 by Student’s t test). **(C)** Wild-type and *Foxd4*-LOF chimeras collected at E9.5, E10.5 and E11.5 with percentage of specimens showing anterior defects. **(D)** *Foxd4* ^*Δ7*/*Δ8*^ E9.5 chimera displays truncated head, stained for SOX1 (red) and DAPI (blue), Maximum intensity z projection (Full panels for immunofluorescence imaging of chimeras are in Supplementary Figure 3). **(E)** The range of head phenotypes in *Foxd4* ^*Δ7*/*Δ8*^ E10.5 chimeras showing neural tube closure defects and craniofacial deformities. Brightfield imaging and dsRed merged image. **(F)** Neural tube closure defect of a representative *Foxd4* ^*Δ7*/*Δ8*^ derived E10.5 chimera, stained for neurofilament (green), TFAP2a (red) and DAPI (blue). Left: Maximum intensity z projection, right: coronal plane through z stacks. **(G)** Craniofacial defect of a representative *Foxd4* ^*Δ7*/*Δ8*^ E10.5 chimera stained for neurofilament (green), SOX2 (red) and DAPI (blue). Left: Maximum intensity z projection, right: coronal plane through z stacks. **(H)** OTX2 expression in the mid-brain and eye is reduced in representative *Foxd4* ^*Δ7*/*Δ8*^ E11.5 chimera embryo (red), Neurofilament staining (green) reveals exencephaly in mutant chimera. Maximum intensity z projection. **(I)** Open neural tube in a representative *Foxd4* ^*Δ7*/*Δ8*^ E11.5 chimera embryo, stained for neurofilament (green), SOX2 (red) and DAPI (blue). Maximum intensity z projection. All scale bars = 500 µm.

To analyze the function of FOXD4 during mouse development, wild-type or *Foxd4*-LOF mESCs were injected into 8-cell host embryos ubiquitously expressing *dsRed* (Figure 3A). Host embryos were injected with either wild-type or *Foxd4* ^*Δ7*/*Δ8*^ mESCs (15 embryos each), chimeric embryos were collected at E8.0 and the relative contribution of mESCs was quantified using fluorescence microscopy. Three chimeras of each genotype with high (>60%) contribution were kept for RNA assay (Supplementary Figure 4A). E8.0 chimeras that showed high contribution of mESCs in fluorescence imaging had significantly lower *dsRed* expression compared to un-injected embryos (Figure 3B). Chimeras with high contribution of *Foxd4* ^*Δ7*/*Δ8*^ mESCs showed significantly reduced *Foxd4* expression compared to wild-type mESC injected chimeras (Figure 3B).

*Foxd4*-LOF chimeric embryos had visible neural tube closure defects and truncated forebrain tissue at E9.5, E10.5 and E11.5, whilst none of the wild-type mESC derived chimeras that were collected displayed an abnormal head phenotype (Figure 3C). At E9.5, 3/13 *Foxd4* ^*Δ7*/*Δ8*^ chimeras displayed anterior defects, compared to 0/9 for wild-type chimeras (Supplementary Figures 5A,B and 6A,B). No anterior defects were evident in E10.5 wild-type chimeras (0/12) (Supplementary Figure 7A,B). 14/18 *Foxd4* ^*Δ7*/*Δ8*^, 2/2 *Foxd4* ^*Δ2*/*Δ2*^, and 2/4 *Foxd4* ^*Δ2*/*Δ1*^ chimeras collected at E10.5 displayed anterior developmental defects (Supplementary Figures 2C,F and 8A,B). Finally, 0/6 wild-type E11.5 chimeras and 2/8 E11.5 *Foxd4* ^*Δ7*/*Δ8*^ chimeras showed anterior defects (Supplementary Figure 9A,B).

*Foxd4* ^*Δ7/Δ8*^ chimeras collected at E9.5 with high contribution had comparable expression of neuroectoderm marker SOX1, though displayed severe truncation of the head tissue compared to wild-type control (Figure 3D, Supplementary Figure 4B). At E10.5 a range of head defect phenotypes were evident in *Foxd4* ^*Δ7/Δ8*^, *Foxd4* ^*Δ2/Δ2*^ and *Foxd4* ^*Δ2/Δ1*^ chimeras (Figure 3E, Supplementary Figure 2C,F). NEFM (neurofilament) staining shows exencephaly in the midbrain and hindbrain, though there were no defects in the caudal neural tube in any *Foxd4*-LOF chimeras (Figure 3F). Craniofacial defects were also common among *Foxd4*-LOF chimeras including truncated facial tissue and abnormal forebrain patterning (Figures 3E,G, Supplementary Figure 4D).

In *Foxd4* ^*Δ7*/*Δ8*^ chimeras collected at E11.5, exencephaly was evident in the rostral neural tube (Figures 3H,I, Supplementary Figures 4E,F). The protein OTX2 that is normally expressed in the midbrain and eyes of E11.5 wild-type embryo, was not detected in *Foxd4* ^*Δ7/Δ8*^ chimeras (Figure 3H). All the defects seen were in anterior head and neural tube tissues, indicating the specific role of FOXD4 in the anterior neural and midline tissue in late gastrulation/early organogenesis.

### FOXD4 Is Required for Anterior Neurectoderm and Neural Crest Specification

**FIGURE 4:**
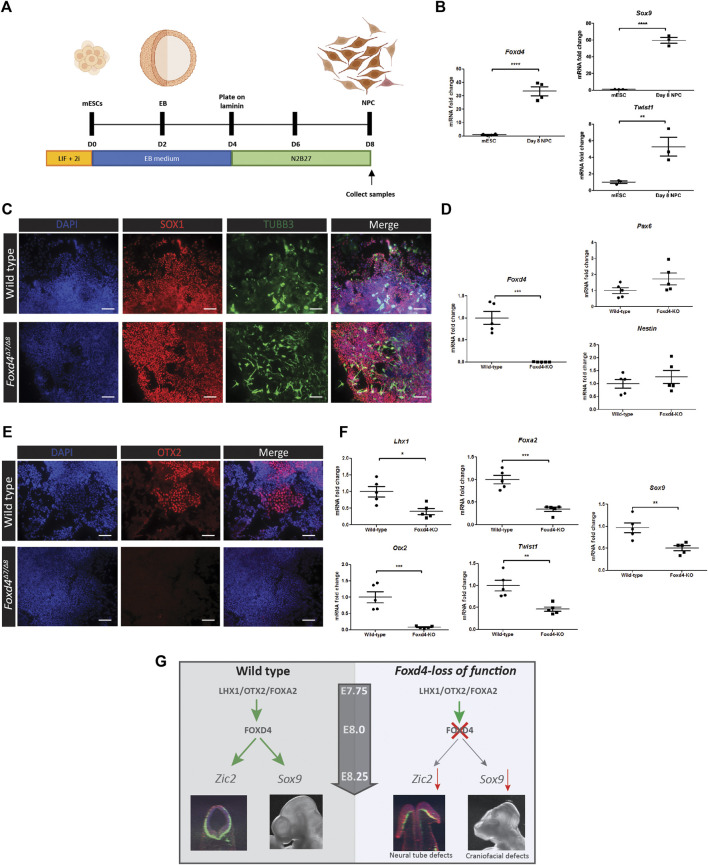
Genes associated with anterior tissues and neural crest cells are downregulated in *Foxd4*-LOF mESCs during neural differentiation. **(A)** Schematics of neural precursor cell differentiation protocol. mESCs were cultured in 2i/Lif media without feeder cells. mESCs are cultured in aggrewells in EB media for 48 h before being placed on shaker plates for a further 48 h. The EBs were then moved onto laminin coated dishes and cultured in N2B27 media for 4 days **(B)** RT-qPCR analysis shows 30-fold increase in expression of *Foxd4* (relative to *Ubc*) neural precursor cells (NPCs) compared to mESCs. *Sox9* and *Twist1* are also highly expressed compared to undifferentiated mESCs (N = . **(C)** Wildtype and *Foxd4* ^*Δ7*/*Δ8*^ NPCs both show strong expression of SOX1 (red) and Tubulin-βIII (green) after 8 days of differentiation. N = 4. Scale bar = 100 µm. **(D)** RT-qPCR analysis shows significantly reduced *Foxd4* expression, though comparable level of expression of *Pax6* and *Nes* (relative to *Ubc*) in *Foxd4* ^*Δ7*/*Δ8*^ NPCs compared to wild-type NPCs. **(E)** *Foxd4* ^*Δ7*/*Δ8*^ NPCs do not express OTX2 (red) after 8 days of differentiation. N = 4. Scale bar = 100 µm. **(F)** RT-qPCR analysis shows reduced expression of *Foxd4*, *Lhx1*, *Otx2, Foxa2*, *Twist1 and Sox9* (relative to *Ubc*) in wild-type vs *Foxd4* ^*Δ7*/*Δ8*^ Day 8 NPCs. Data are presented as mean ± SEM of four independent treatments (*p < 0 0.05, **p < 0 0.01, ***p < 0 0.001 by Student’s t test). **(G)** Schematic: In the anterior tissue of the E7.75 mouse embryo, *Foxd4* expression is controlled by the LHX1/OTX2/FOXA2 TF complex. FOXD4 is then required for regulating the expression of *Zic2* in the ANE for neurulation. The loss of FOXD4 function in the neuroectoderm leads deficiency of cranial neural crest cells, revealed by the reduced expression of *Sox9* and craniofacial defects in *Foxd4*-LOF chimeric embryos.

We adapted a protocol from Varshney et al. (2017) for the differentiation of mESCs to neural precursor cells (NPC) (Figure 4A), revealed by high levels of *Foxd4* expression in wild-type NPCs at Day 8 of culture (Figure 4B). NCC markers *Twist1* and *Sox9* were also highly expressed in the NPCs compared to undifferentiated mESCs (Figure 4B). Both wild-type and *Foxd4* ^*Δ7/Δ8*^ mESCs expressed a high level of neural ectoderm marker SOX1 and neuron specific Class III *β*-tubulin (TUBB3) (Figure 4C). *Foxd4* ^*Δ7/Δ8*^ day 8 NPCs had significantly reduced *Foxd4* mRNA expression compared to the wild-type (Figure 4D). mRNA expression of neurectoderm markers *Pax4* and *Nestin (Nes)* were not significantly different in *Foxd4* ^*Δ7/Δ8*^ NPCs (Figure 4D). In contrast, the head organizer genes *Otx2, Lhx1* and *Foxa2* were significantly reduced (Figures 4E,F). The loss of *Otx2* expression is consistent with the reduction in OTX2 expression seen in the *in vivo* model (Figure 3H). NCC markers *Twist1* and *Sox9* were also downregulated in *Foxd4* ^*Δ7/Δ8*^ NPCs (Figure 4F) compared to wild-type, though our scRNA-seq analysis did not show significant co-expression of *Foxd4* and *Twist1* at E7.75 or E8.25 (Figure 1B).

## Discussion

Our study has revealed a novel role of FOXD4 in the development of the embryonic head and neural tube in mouse embryos. Single cell transcriptomic analysis confirms published spatial RNA expression pattern in E7.5-E7.75 embryos showing *Foxd4* expression in the anterior midline and anterior neurectoderm tissues (Kaestner et al., 1995). The anterior midline cell population at E7.75 marked by *Lhx1* and *Foxa2* are the precursors of the anterior mesendoderm underlying neuroectoderm of the head folds (Kinder et al., 2003). Co-expression of *Foxd4* with *Lhx1* and *Foxa2* in the anterior midline (notochord) and definitive endoderm populations at E7.75 imply a shared mechanism of these transcription factors in these tissues. Reduced *Foxa2* or *Lhx1* expression in the anterior embryo has been shown to ablate *Foxd4* expression in the same region (Tamplin et al., 2008; Sibbritt et al., 2018). Furthermore, ChIP-seq data show LHX1 and FOXA2 binding sites upstream of *Foxd4*. Our *in vitro* results show that a loss of FOXD4 activity also reduces the expression of *Lhx1* and *Foxa2*. This shared relationship may indicate that all three transcription factors act together to define the anterior midline tissue of the late gastrulation stage mouse embryo.

For neural induction of the anterior epiblast, firstly the anterior visceral endoderm (AVE) establishes the adjacent neurectoderm, then the AME acts to maintain the neurogenic differentiation (reviewed in Martinez-Barbera and Beddington, 2003). *Foxd4* is not expressed highly in the AVE; its expression peaks at E8.0-E8.25 in anterior neurectoderm where it is co-expressed with two other anterior neurectoderm marker genes *Otx2* and *Hesx1* (Figure 1D). *Hesx1* and *Otx2* are expressed in the forebrain and midbrain of the developing mouse embryos and mutations in each of these transcription factors result in truncated or deficient head tissues (Matsuo et al., 1995; Martinez-Barbera et al., 2000). In *Foxd4* ^*Δ7/Δ8*^ neuruloids, *Hesx1* expression is significantly reduced and similarly there is no OTX2 protein expression in *Foxd4* ^*Δ7/Δ8*^ neural precursor cells. These findings indicate that FOXD4 is essential in the anterior neurectoderm tissues of the late gastrula stage mouse embryo. An evident phenotype of *Foxd4*-LOF mESC derived chimeras is the reduced head size and forebrain defect. This phenotype coupled with reduced expression of OTX2 in E11.5 *Foxd4* ^*Δ7/Δ8*^ chimeras show that FOXD4 is required for the development of anterior neurectoderm in mouse embryos.

In *Xenopus* embryos, the homolog of *Foxd4* (*Foxd4/5*) is crucial for the induction and maintenance of neurectoderm cells at gastrula and neural plate stage of development (Yan et al., 2009; Neilson et al., 2012). The AB domain in the N-terminal FOXD4/5 protein was shown to upregulate the immature neural precursor marker *Zic2,* and the mouse, FOXD4 has homologous activity when expressed in *Xenopus* embryos (Sherman et al., 2017). In an *in vitro Foxd4-*LOF neuruloid model, the expression of *Zic2* is also significantly down-regulated, consistent with literature showing that a loss *Zic2* function in the embryo leads to neural tube defects (Warr et al., 2008). Despite a reduction in *Zic2* expression, closure defects were only found in the rostral neural tube *Foxd4*-LOF mESC derived chimeras. The expression of *Zic2* in the caudal neural tube may therefore be influenced by other factors such as PAX3 or CDX2 (Zhao et al., 2014). It is likely that a primary function of FOXD4 is to regulate *Zic2* activity in the anterior neurectoderm to enable proper neural tube closure (Figure 4G). Contrary to a previous study in mESCs (Sherman et al., 2017), we found FOXD4 is not needed to generate neural precursor cells *in vitro*. *Foxd4* ^*Δ7/Δ8*^ neural precursor cells (NPCs) express neural precursor markers SOX1, TUBB3, *Pax6* and *Nes* at levels equivalent to wild-type NPCs. Likewise, in *Foxd4*-LOF chimeras, neurofilament is expressed at levels similar to wild type, although the pattern of innervation is disrupted.

A closely related protein to FOXD4; FOXD3, also contains acidic, forkhead and Eh1 domains (Wijchers et al., 2006) and has been demonstrated to be essential for NCC specification and maintenance of neural crest progenitor cells (Dottori et al., 2001; Teng et al., 2008). Our *in vitro* models also indicate that FOXD4 is driving early NCC specification. The NCCs are a migratory population of cells that arise firstly at border of neural plate and non-neural ectoderm cells (Wang et al., 2011). After gastrulation, cranial neural crest (CNC) cells delaminate from the dorsal neural tube and begin to express CNC specific markers including *Sox9* and *Twist1* (Mori-Akiyama et al., 2003; Soldatov et al., 2019). In the *Foxd4* ^*Δ7/Δ8*^ neuruloids and NPCs, *Sox9* transcripts are significantly downregulated compared to wild-type controls. Similarly, RNA expression of neural crest-related gene, *Twist1*, was reduced in *Foxd4* ^*Δ7/Δ8*^ NPCs. Our scRNA-seq analysis also shows co-expression of *Foxd4* and *Sox9* in CNC precursor populations of the rostral neurectoderm. We therefore propose that FOXD4 is regulating the expression of *Sox9* in the CNC progenitor population and has a shared function alongside FOXD3 to specify CNC cells. A loss of FOXD4 activity may be affecting the allocation of the CNC precursor population and further impacts on the pattern of cranial nerve innervation of the head tissues (Figure 4G). These data indicate that FOXD4 is not required for the specification of neuronal cell lineages but is required for the differentiation of the head tissues and specification of CNC tissue.

## Conclusion

Our study has revealed that FOXD4 acts in conjunction with LHX1, FOXA2, OTX2, and HESX1 to regulate the activity of key genes associated with neural tube morphogenesis and CNC specification in the anterior midline tissue and the anterior neurectoderm tissues. Further study of the transcriptional targets of FOXD4 in the neuroectoderm and neural crest cells will shed more light on the pleiotropic role of this transcription factor in craniofacial development.

## Data Availability

All the code for data analysis is publicly available at https://github.com/naryamanesh/Pijuan_Foxd4. All the mESC and XEN cell lines generated for this article are freely available to the scientific community upon request.

## Supplementary Material

Supplementary Figure S1
**(A)** Uniform Manifold Approximation and Projection for Dimension Reduction (UMAP) for individual cells at E7.75 and E8.25 (data from Pijuan-Sala et al, 2019). Colours represent the relevant cell types expressing the genes of interest: *Foxd4*, *Lhx1* and *Foxa2* at E7.75 and *Foxd4, Otx2* and *Hesx1* at E8.25. *Foxd4* at E7.75 shows highest expression in notochord, neurectoderm and definitive endoderm. *Foxd4* at E8.25 shows highest expression in definitive endoderm, notochord, forebrain/midbrain/hindbrain and rostral neurectoderm. **(B)** UMAP for individual cells at E7.75 and E8.25 (data from Pijuan-Sala et al. (2019). Colours represent the relevant cell types expressing the genes of interest. Co-expression data of *Foxd4* with *Zic1*, *Zic2*, *Twist1* or *Sox9* are shown in red. Co-expression of *Foxd4*/*Zic2* and *Foxd4*/*Sox9* are mainly in the rostral neurectoderm at E7.75 and forebrain/midbrain/hindbrain in E8.25 embryos.

Supplementary Figure S2
**(A)** Schematic representation of CRISPR-Cas9 editing on *Foxd4* for clone *Foxd4* ^*Δ2*/*Δ2*^. CRISPR-Cas9 gRNA 1 targeted the DNA sequence corresponding to the N-terminal AB domain of *Foxd4,* resulting in 2bp deletions in each allele. **(B)** Relative fluorescence intensity of host derived tissue (dsRed.T3) over area for each *Foxd4* ^*Δ2*/*Δ2*^ chimera collected at E10.5 (compared to un-injected E10.5 embryos). **(C)** Brightfield and dsRed.T3 imaging of E10.5 *Foxd4* ^*Δ2*/*Δ2*^ chimeras ranked from least mESC contribution to most. Arrows indicate anterior defect. **(D)** Schematic representation of CRISPR-Cas9 editing on *Foxd4* for clone *Foxd4* ^*Δ2*/*Δ1*^. CRISPR-Cas9 gRNA 2 targeted the DNA sequence corresponding to the region between the AB and forehead domains of *Foxd4,* resulting in a 2bp deletion and 1bp insertion in each allele. **(E)** Relative fluorescence intensity of host derived tissue (dsRed.T3) over area for each *Foxd4* ^*Δ2*/*Δ1*^ chimera collected at E10.5 (compared to un-injected E10.5 embryos). **(F)** Brightfield and dsRed.T3 imaging of E10.5 *Foxd4* ^*Δ2*/*Δ1*^ chimeras ranked from least mESC contribution to most. Arrows indicate anterior defect. All scale bars = 500µm

Supplementary Figure S3
**(A)** Immunofluorescence imaging of extraembryonic endoderm (XEN) cells derived from *dsRed*-expressing E3.5 mouse blastocysts. XEN cells show expression of endoderm markers FOXA2 and SOX17 but not pluripotency factor OCT4. N=4. Scale bar = 50 µm. **(B)** RT-qPCR analysis shows expression of endoderm genes, *Foxa2* and *Sox17*, extraembryonic endoderm gene *Gata6* and pluripotency factor, *Sox2* (relative to *Actb*) in dsRed-positive XEN cells compared to R1 mESCs. N=1. **(C)** Neuruloids showing dsRed-expressing XEN cells localized on the outside of the neuruloids, while the ESCs colonized the core. Wild-type neuruloids show SOX1 expression. Single z-stack. N=4. Scale bar = 300 μm. **(D)** RT-qPCR analysis shows increased expression of anterior tissue genes, *Lhx1, Otx2, Foxd4* and *Hesx1* (relative to *Actb*) in Day 4 neuruloids compared to conventional Day 4 embryoid bodies (EBs). N=1. **(E)** 96 hour differentiated wild-type neuruloids show expression of early neural marker SOX1. Maximum intensity z projection. N= 4. Scale bar = 300 μm.

Supplementary Figure S4
**(A)** Brightfield and dsRed imaging of E8.0 dsRed.T3 embryos and E8.0 chimeras with either wild-type or *Foxd4* ^*Δ7*/*Δ8*^ mESCs. **(B)** Wild-type and *Foxd4* ^*Δ7*/*Δ8*^ E9.5 chimeras stained for SOX1 (red). *Foxd4* ^*Δ7*/*Δ8*^ chimera displays truncated head. Maximum intensity z projection. **(C)** Neural tube defect of representative *Foxd4* ^*Δ7*/*Δ8*^ E10.5 chimera embryo stained for neurofilament (green), TFAP2a (red) and DAPI (blue). Maximum intensity z projection. **(D)** Craniofacial defect of representative *Foxd4* ^*Δ7*/*Δ8*^ E10.5 chimera embryo stained for neurofilament (green), SOX2 (red) and DAPI (blue). Maximum intensity z projection. **(E)** Mid-brain/eye marker OTX2 expression is reduced in representative *Foxd4* ^*Δ7*/*Δ8*^ derived E11.5 chimera embryo (red), neurofilament staining (green) reveals exencephaly in mutant chimera. Maximum intensity z projection. **(F)** Exencephaly shown in representative *Foxd4* ^*Δ7*/*Δ8*^ derived E11.5 chimera embryo, stained for neurofilament (green), SOX2 (red) and DAPI (blue). Maximum intensity z projection. Scale bar = 500 µm

Supplementary Figure S5
**(A)** Relative fluorescence intensity of host derived tissue (dsRed.T3) over area for each wild-type chimera collected at E9.5 (compared to un-injected E9.5 embryos). **(B)** Brightfield and dsRed.T3 imaging of E9.5 wild-type chimeras ranked from low mESC contribution to high. All scale bars = 500µm

Supplementary Figure S6
**(A)** Relative fluorescence intensity of host derived tissue (dsRed.T3) over area for each *Foxd4* ^*Δ7*/*Δ8*^ chimera collected at E9.5 (compared to un-injected E9.5 embryos). **(B)** Brightfield and dsRed.T3 imaging of E9.5 *Foxd4* ^*Δ7*/*Δ8*^ chimeras ranked from low mESC contribution to high. Arrows indicate anterior defect. All scale bars = 500 µm

Supplementary Figure S7
**(A)** Relative fluorescence intensity of host derived tissue (dsRed.T3) over area for each wild-type chimera collected at E10.5 (compared to un-injected E10.5 embryos). **(B)** Brightfield and dsRed.T3 imaging of E10.5 wild-type chimeras ranked from low mESC contribution to high. All scale bars = 500 µM

Supplementary Figure S8
**(A)** Relative fluorescence intensity of host derived tissue (dsRed.T3) over area for each *Foxd4* ^*Δ7*/*Δ8*^ chimera collected at E10.5 (compared to un-injected E10.5 embryos). **(B)** Brightfield and dsRed.T3 imaging of E10.5 *Foxd4* ^*Δ7*/*Δ8*^ chimeras ranked from low mESC contribution to high. Arrows indicate anterior defect. All scale bars = 500 µM

Supplementary Figure S9
**(A)** Relative fluorescence intensity of host derived tissue (dsRed.T3) over area for each wild-type chimera collected at E11.5 (compared to un-injected E11.5 embryos). **(B)** Relative fluorescence intensity of host derived tissue (dsRed.T3) over area for each *Foxd4* ^*Δ7*/*Δ8*^ chimera collected at E11.5 (compared to un-injected E11.5 embryos). **(C)** Brightfield and dsRed.T3 imaging of E11.5 wild-type chimeras ranked from low mESC contribution to high. **(D)** Brightfield and dsRed.T3 imaging of E11.5 *Foxd4* ^*Δ7*/*Δ8*^ chimeras ranked from low mESC contribution to high. Arrows indicate anterior defect. All scale bars = 500µM
