# Supplementary material for: Loss of Foxd4 Impacts Neurulation and Cranial Neural Crest Specification During Early Head Development
Source: Front Cell Dev Biol. 2022 Feb 1;9:777652. doi: 10.3389/fcell.2021.777652 (PMC8843869; doi:10.3389/fcell.2021.777652)
Supplement: Supplementary file 1 [file Table1.docx]

**Supplementary Table S1**

| **Gene** | **Forward primer (5’-)** | **Reverse primer (5’-)** | **Source** |
| --- | --- | --- | --- |
| *Actb* | AGCACCCTGTGCTGCTCA | GTACGACCAGAGGCATACA | (Fossat et al., 2011) |
| *Ubc* | CGTCGAGCCCAGTGTTACCACCAAGAAGG | CCCCCATCACACCCAAGAACAAGCACAAG | (Barratt et al., 2018) |
| *Foxd4* | AGTTCCGACCAGGATGAGGT | GAGGAACCCGGAGGAGTTAC | Primer Blast |
| *Foxa2* | GAGTTAAAGTATGCTGGGAGC | TTGCTCACGGAAGAGTAGC | Primer Blast |
| *Lhx1* | AAGCAACTGGAGACGTTGAA | CTAGCGCGCTTAGCTGTTT | (Sibbritt et al., 2018) |
| *Otx2* | ccaaatctacccaccaagga | agagcttccagaacgtcgag | (Sibbritt et al., 2018) |
| *Hesx1* | AGCATTTTAGGACTGGACCA | ATGAAGTCTCACTGGGAAGA | (Sibbritt et al., 2018) |
| *Zic1* | GCAAGATGTGCGATAAGTCC | GGTTGTCTGTTGTGGGAGAC | (Barratt et al., 2018) |
| *Zic2* | TCGTTGCGGAAGCACATGAA | ACAGGTTGGAGCTGCTTTGT | (Barratt et al., 2018) |
| *Sox2* | ATGAACGGCTGGAGCAAC | ATGTAGGTCTGCGAGCTGGT | Primer Blast |
| *Sox9* | AGGAAGTCGGTGAAGAACGG | GGACCCTGAGATTGCCCAGA | Primer Blast |
| *dsRed.t3* | CCCCGTAATGCAGAAGAAGA | GGTGATGTCCAGCTTGGAGT | (Phua et al., 2013) |
| *Nes* | CCAGAGCTGGACTGGAACTC | ACCTGCCTCTTTTGGTTCCT | Primer Blast |
| *Pax6* | TAACGGAGAAGACTCGGATGAAGC | CGGGCAAACACATCTGGATAATGG | Primer Blast |
