# Supplementary figures and images for: Loss of Foxd4 Impacts Neurulation and Cranial Neural Crest Specification During Early Head Development
Source: Front Cell Dev Biol. 2022 Feb 1;9:777652. doi: 10.3389/fcell.2021.777652 (PMC8843869; doi:10.3389/fcell.2021.777652)

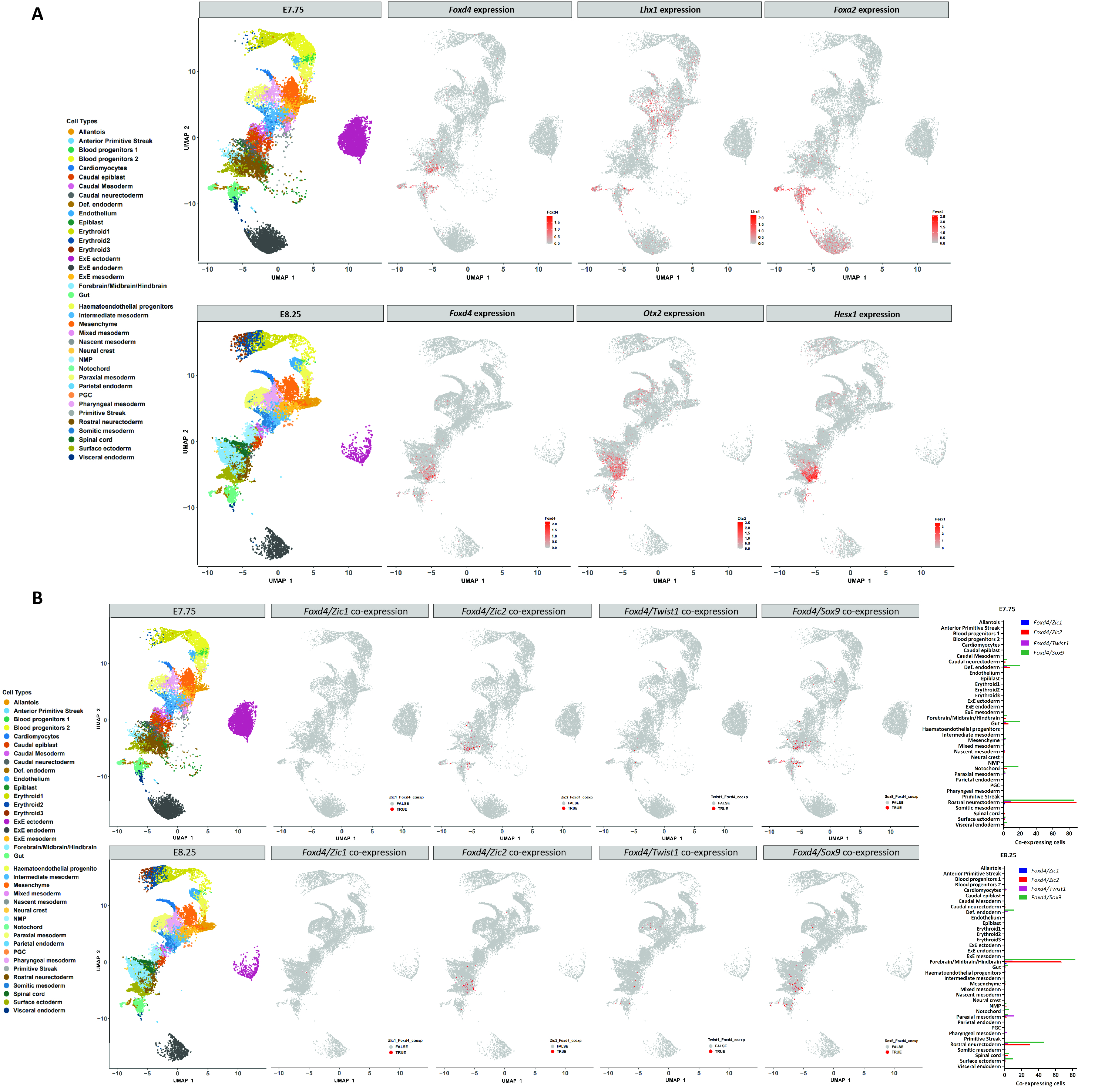

Supplement: Supplementary file 2 [file DataSheet1.zip › Supplementary Figure 1.jpg]

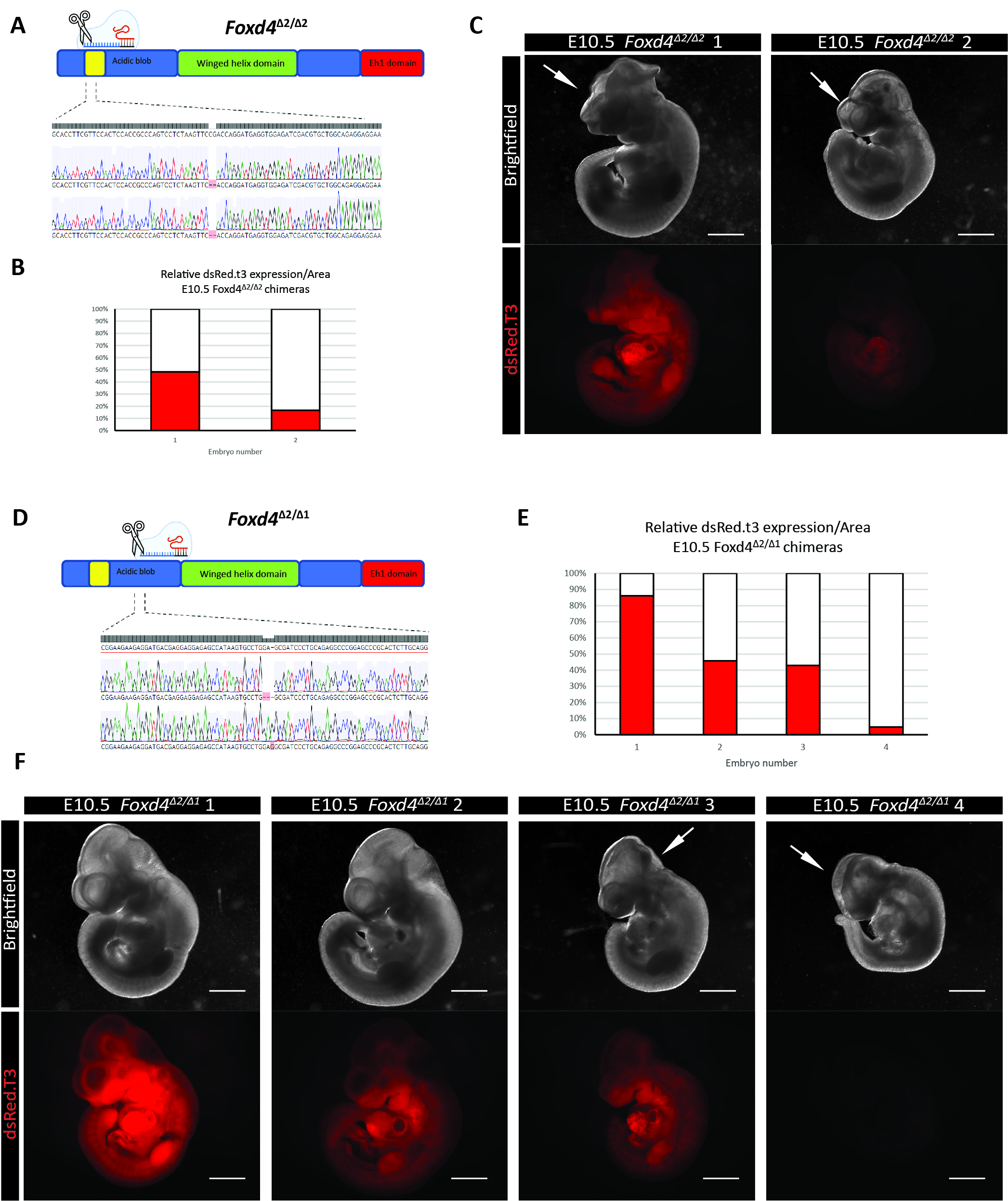

Supplement: Supplementary file 2 [file DataSheet1.zip › Supplementary Figure 2.jpg]

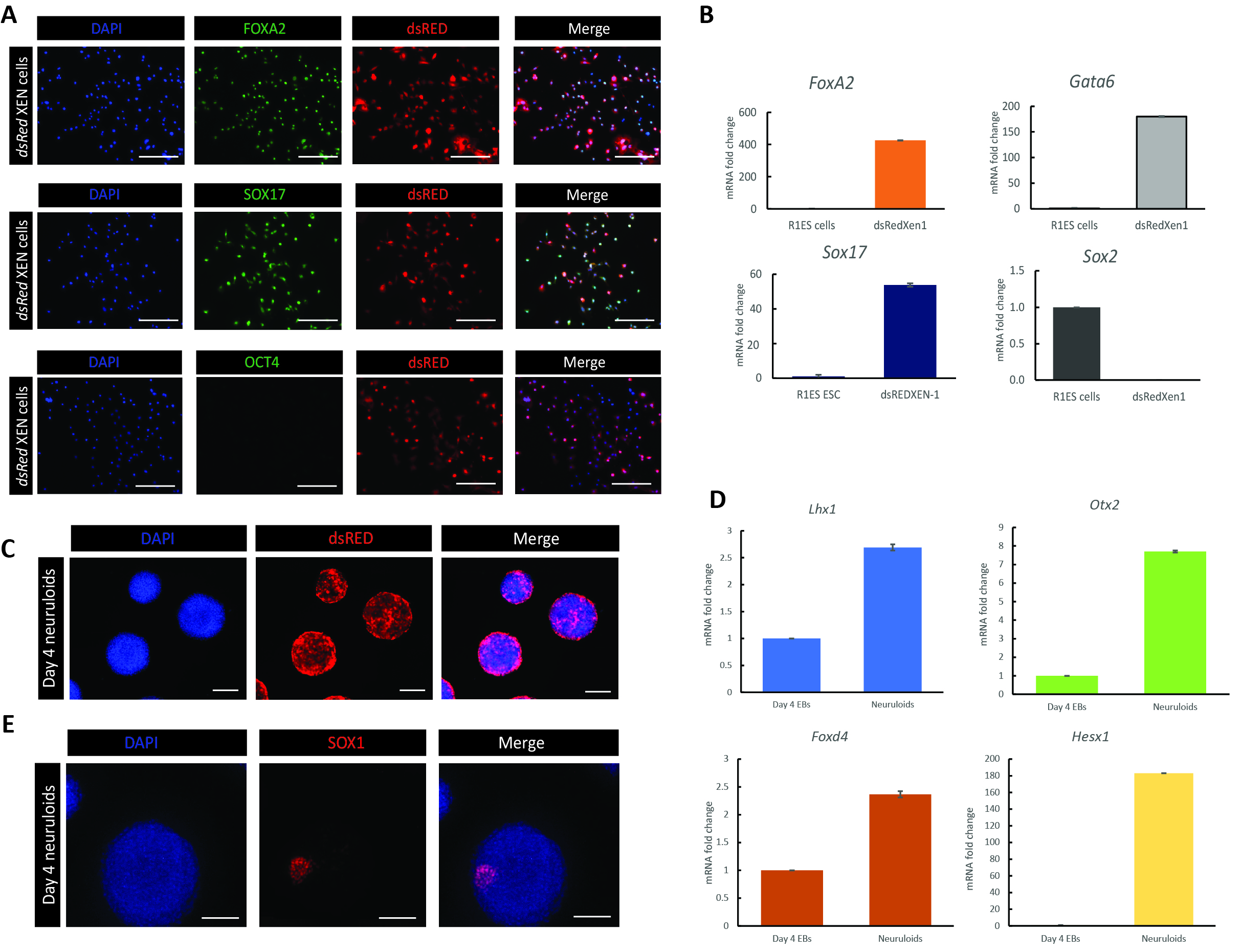

Supplement: Supplementary file 2 [file DataSheet1.zip › Supplementary Figure 3.jpg]

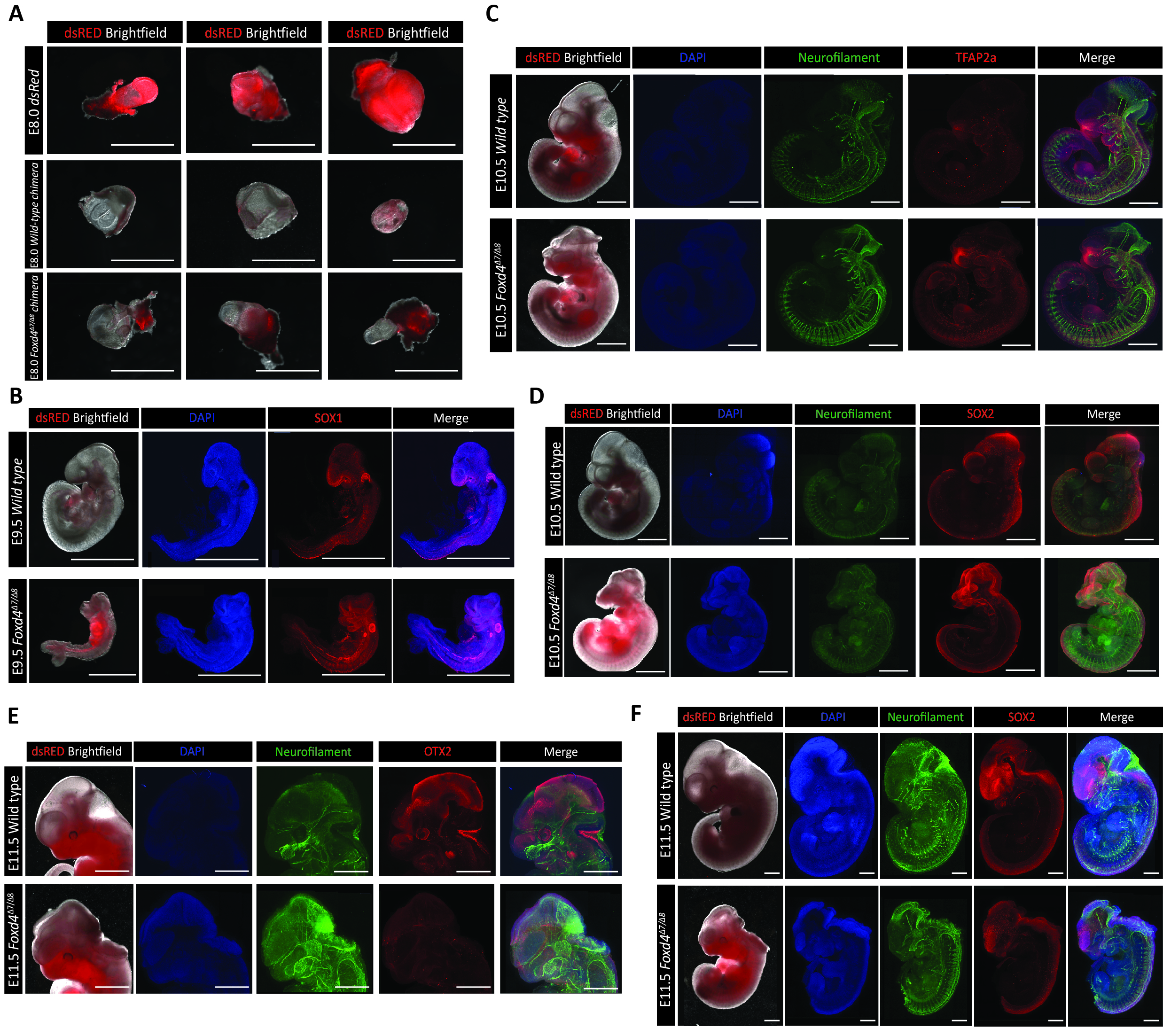

Supplement: Supplementary file 2 [file DataSheet1.zip › Supplementary Figure 4.jpg]

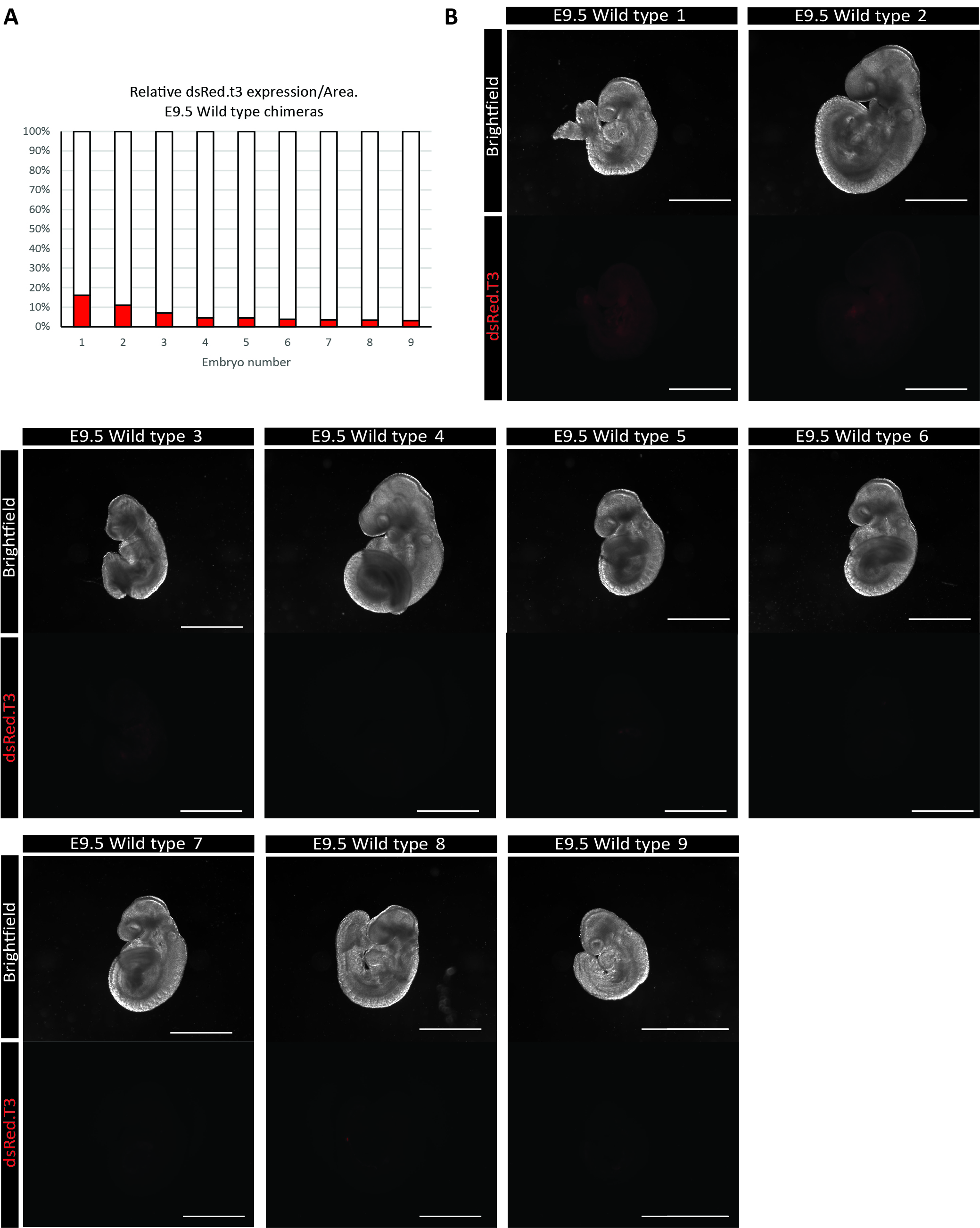

Supplement: Supplementary file 2 [file DataSheet1.zip › Supplementary Figure 5.jpg]

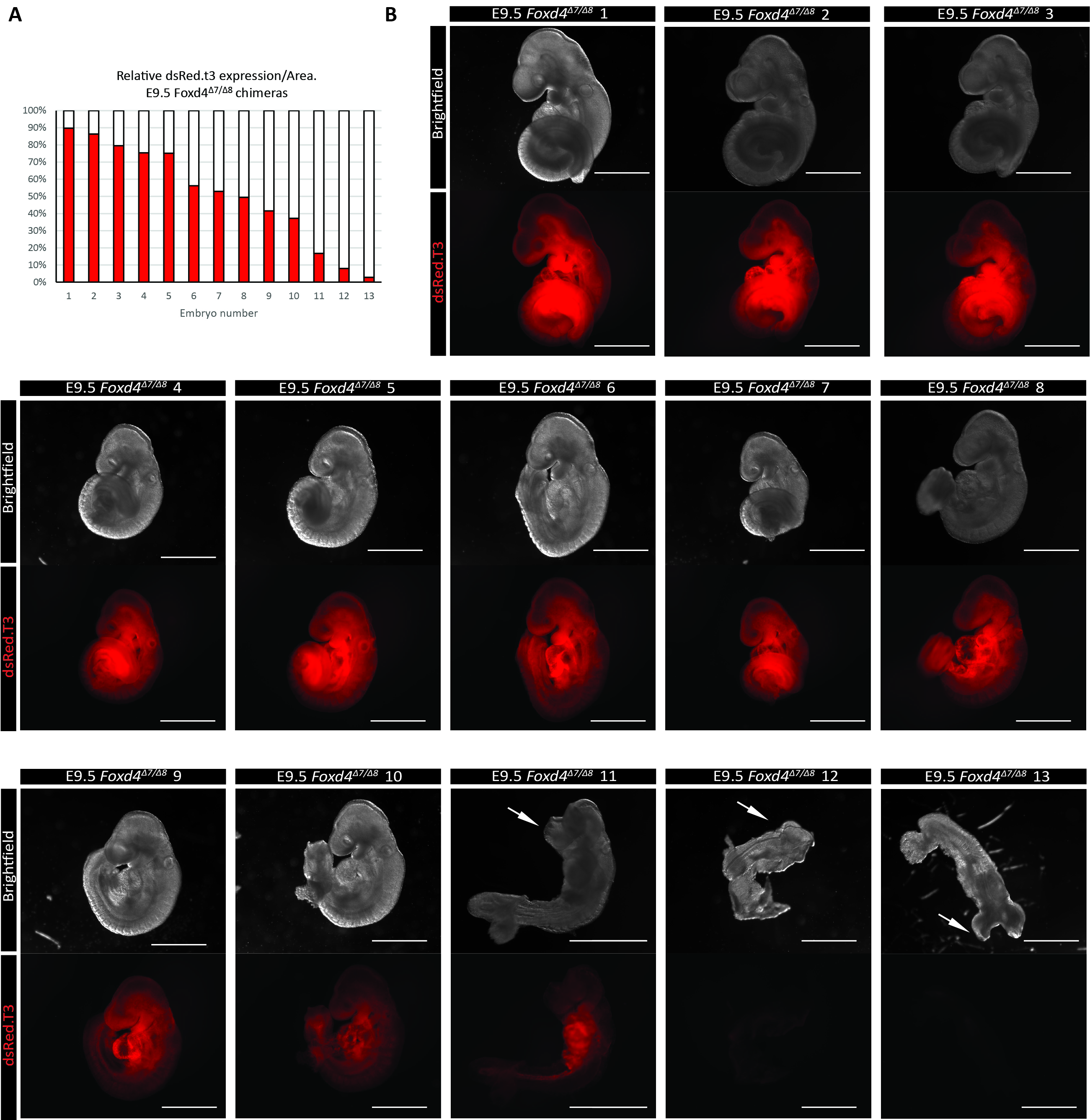

Supplement: Supplementary file 2 [file DataSheet1.zip › Supplementary Figure 6.jpg]

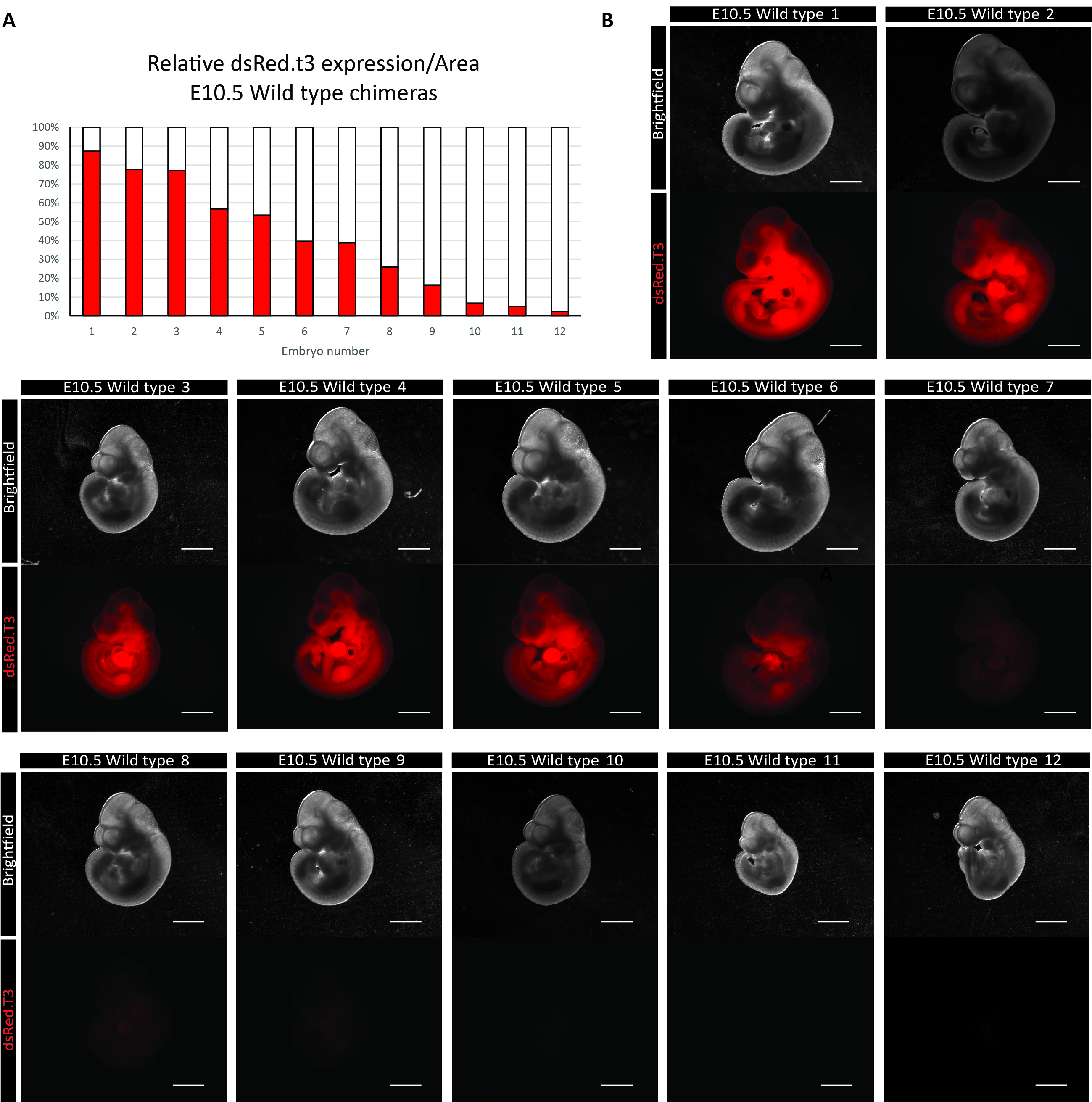

Supplement: Supplementary file 2 [file DataSheet1.zip › Supplementary Figure 7.jpg]

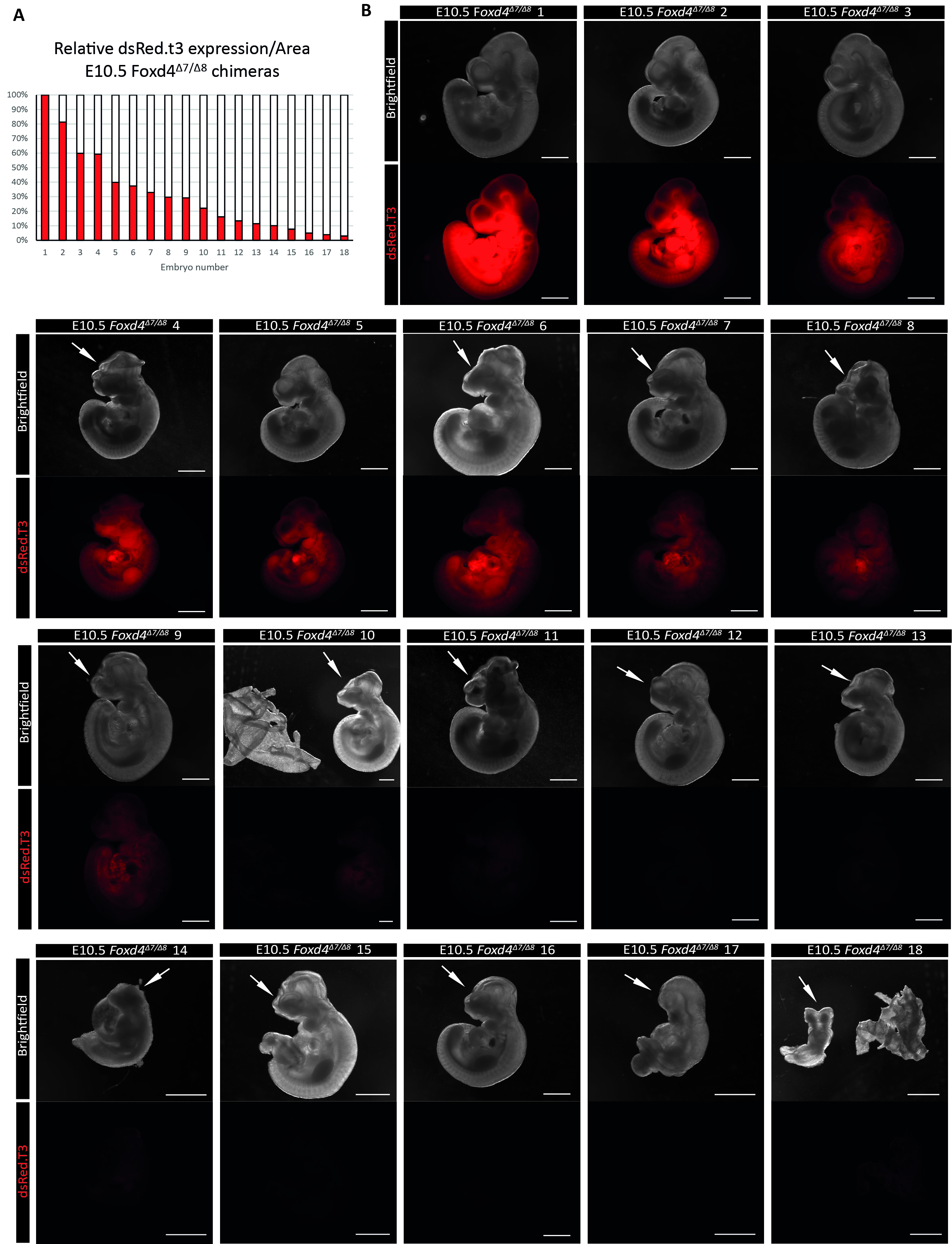

Supplement: Supplementary file 2 [file DataSheet1.zip › Supplementary Figure 8.jpg]

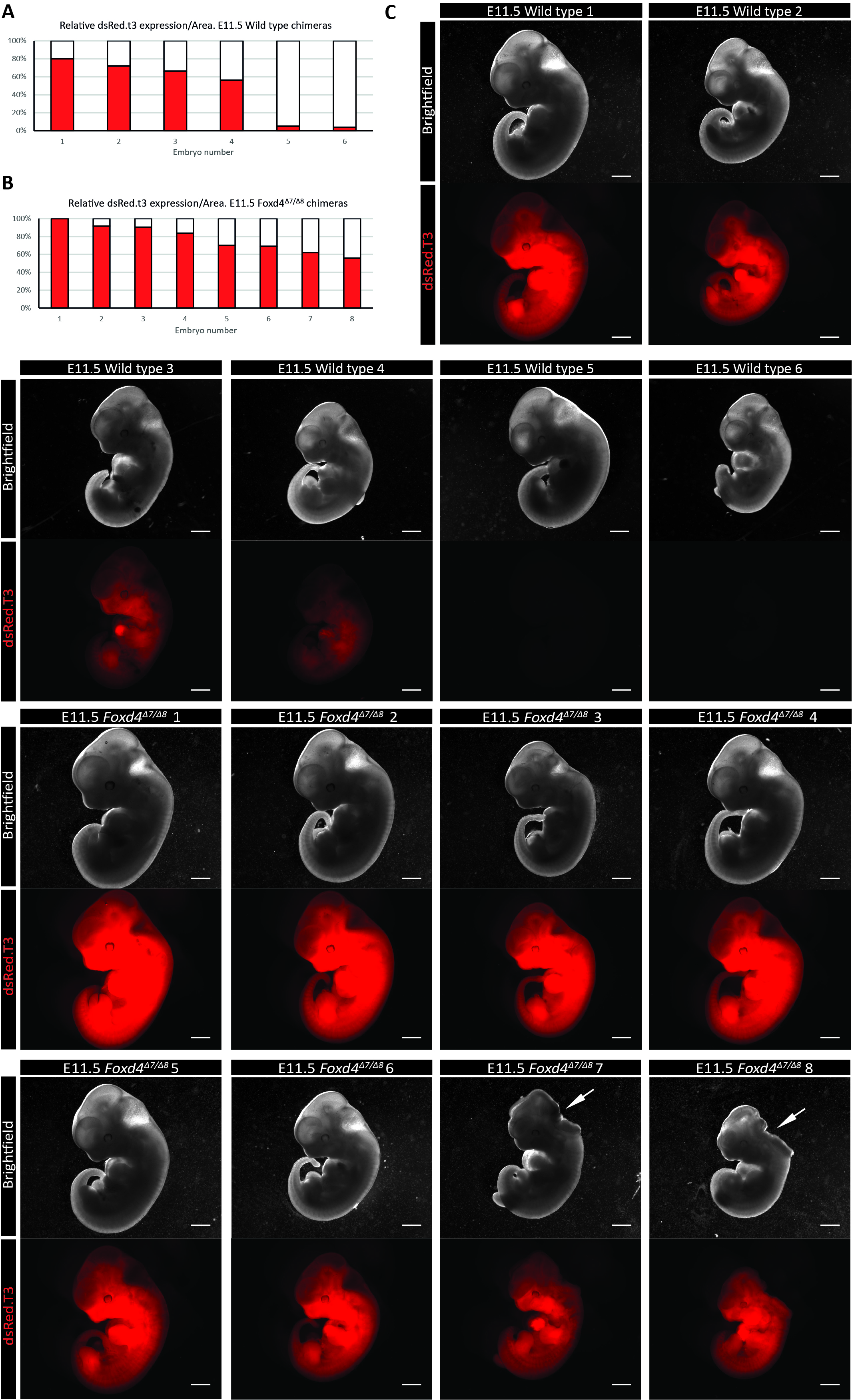

Supplement: Supplementary file 2 [file DataSheet1.zip › Supplementary Figure 9.jpg]
