# Supplementary material for: Loss of Foxd4 Impacts Neurulation and Cranial Neural Crest Specification During Early Head Development
Source: Front Cell Dev Biol. 2022 Feb 1;9:777652. doi: 10.3389/fcell.2021.777652 (PMC8843869; doi:10.3389/fcell.2021.777652)
Supplement: Supplementary file 3 [file Table2.docx]

**Supplementary Table S2**

| **Target** | **Species** | **Source** | **Identifier** | **Concentration used** |
| --- | --- | --- | --- | --- |
| α-SOX1 | Rabbit | Cell Signaling Technology | Cat #4194 | 1:500 |
| α-SOX2 | Rabbit | Abcam | Cat# ab59776 | 1:1000 |
| α-TFAP2A | Rabbit | Abcam | Cat# ab52222; | 1:1000 |
| α-neurofilament | Mouse | DSHB | Cat# F1804 | 1:1000 |
| α-OTX2 | Rabbit | Abcam | Cat# 21990 | 1:250 |
| α-TUBB3 | Mouse | R&D Systems | Cat #MAB1195 | 1:100 |
| α-OCT4 | Rabbit | Abcam | Cat #ab19857 | 1:1000 |
